# Biomass carbon mining to develop nature-inspired materials for a circular economy

**DOI:** 10.1016/j.isci.2023.106549

**Published:** 2023-03-31

**Authors:** Anna Bachs-Herrera, Daniel York, Tristan Stephens-Jones, Ian Mabbett, Jingjie Yeo, Francisco J. Martin-Martinez

**Affiliations:** 1 Department of Chemistry, Swansea University, Swansea SA2 8PP, UK; 2 Sibley School of Mechanical and Aerospace Engineering, Cornell University, Ithaca, NY 14853, USA

**Keywords:** Energy resources, Biotechnology, Biomass, Materials science

## Abstract

A transition from a linear to a circular economy is the only alternative to reduce current pressures in natural resources. Our society must redefine our material sources, rethink our supply chains, improve our waste management, and redesign materials and products. Valorizing extensively available biomass wastes, as new carbon mines, and developing biobased materials that mimic nature’s efficiency and wasteless procedures are the most promising avenues to achieve technical solutions for the global challenges ahead. Advances in materials processing, and characterization, as well as the rise of artificial intelligence, and machine learning, are supporting this transition to a new materials’ mining. Location, cultural, and social aspects are also factors to consider. This perspective discusses new alternatives for carbon mining in biomass wastes, the valorization of biomass using available processing techniques, and the implementation of computational modeling, artificial intelligence, and machine learning to accelerate material’s development and process engineering.

## Graphical abstract

**Figure:**
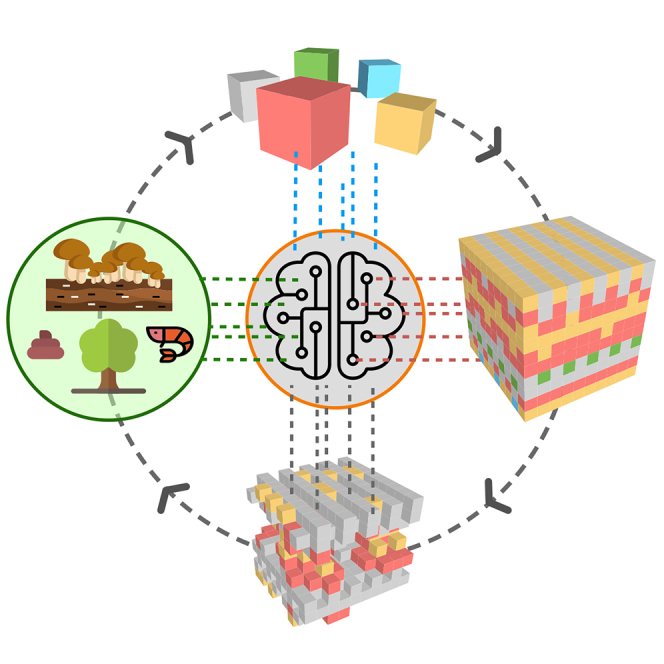

## Introduction

In a world with limited resources, and global challenges derived from the increasing global population, a transition from a linear to a circular economy is mandatory to guarantee a sustainable future for the generations to come. The depletion of fossil fuels and a daily increasing demand for energy and petroleum-derived materials are not compatible, not to mention the devastating consequences on Earth’s climate.^1^ From systems level to the nanoscale, our society must rethink and redefine our raw material sources and supply chains, our waste management, and our materials and product design. The complexity of this transition is extremely high, and all different stakeholders must be considered, including policymakers, investors, manufacturers, educators, researchers, engineers, consumers, and users. From a material engineering perspective, taking advantage of the extensively available biomass wastes, as new carbon mines, and mimicking nature’s efficiency and ability to design out waste is the most promising avenue to achieve high-performance technical solutions for the global challenges ahead.

A plethora of applications that valorize biomass, and implement biomass-derived materials (i.e., biobased), are being intensively investigated, e.g., biofuels, polymers, composites, adhesives, foams, adsorbents, carbon fibers, coatings, supercapacitors, batteries, fuel cells, and biochemicals, just to mention a few.^2^ This is changing the way we think about materials flows, and it is initiating a shift from the traditional linear economy of materials utilization, i.e., take, use, and dispose, to a new biobased economy, as one of the enablers of a wider circular one. One of the greatest challenges for these nature-inspired biobased materials is to provide similar or better performance to their petroleum-derived counterparts, while mimicking nature in diminishing the negative impact on health and environment, including the emission of greenhouse gases. For the past 25 years, some efforts have been made in the reuse, valorization, and utilization of biomass, mostly in the bioenergy sector,^3^^,^^4^ and more recently in the use of biobased molecules.^5^

**Figure 1:**
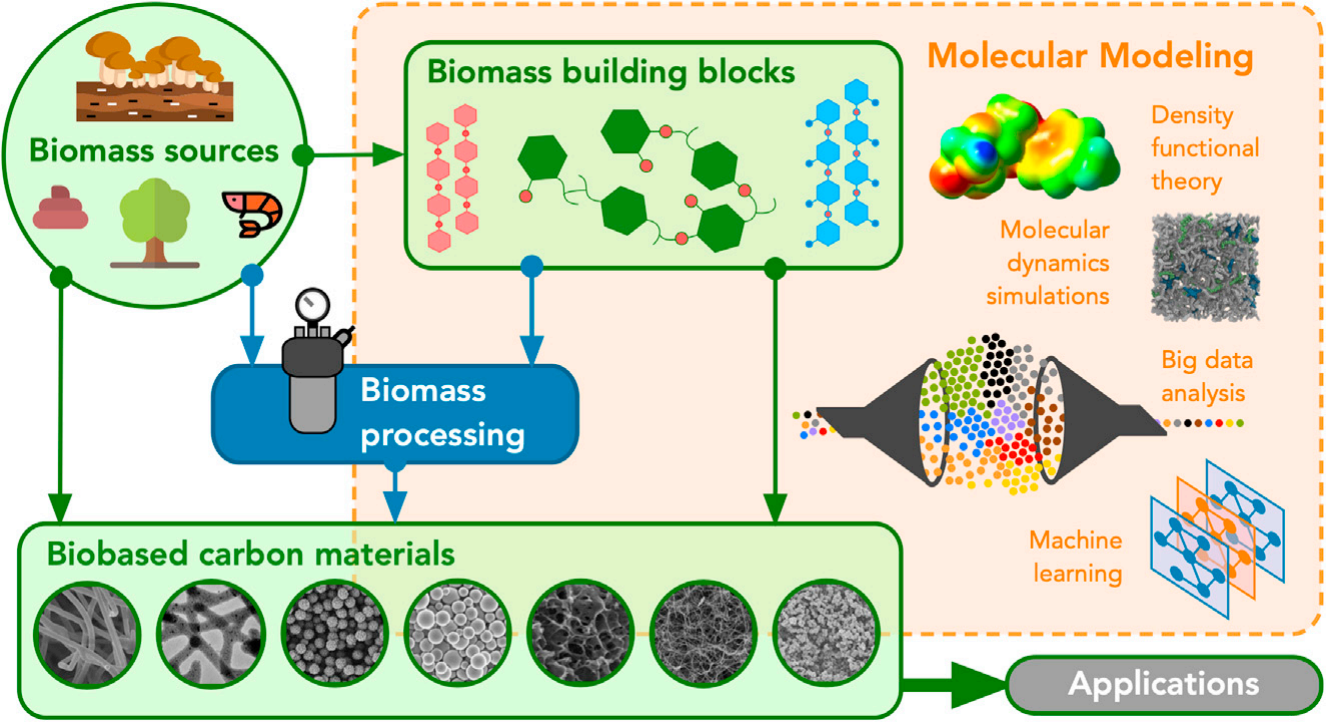
Schematic representation of material flows in carbon mining and valorization from biomass waste, including some of the computational techniques that assist the development of nature-inspired biobased materials. Biomass materials and their building blocks are currently being used for several applications, either as raw materials or after processing. Multiscale modeling, big data analysis, and ML are assisting, among other computational techniques, in the design of these nature-inspired materials.

Furthermore, the use of artificial intelligence (AI), and machine learning (ML), in combination with cloud supercomputing, and high-throughput computational modeling, is accelerating materials discovery and design and therefore contributing to this transition to a new nature-inspired *computational* materials mining that reduces the current pressures on natural resources (Figure 1). However, a more complete integration of these computational tools into the material design phase and into the process engineering of biomass conversion is still a pending task, mostly due to the intrinsic limitations of the models and computational capabilities. More importantly, while AI methods are starting to assist with the discovery of biobased molecules and materials, their use needs to be taken with responsibility and as a complementary but not substitutive alternative to the state-of-the-art methods and experimental procedures. A responsible AI, which considers technical, sociological, and ethical aspects, is required.

A sticking point has been the estimation of biomass resources available. A lack of well-defined criteria for classifying biomass types and composition, as well as for quantifying available biomass sources, is a pending task where big data analysis tools and AI can contribute. Some sources claim that total biomass production is around 140 Gt worldwide,^3^ although according to the United Nations Environment Program this amount only includes agriculture biomass waste.^6^ Other sources provide figures that focus on just one type of biomass waste, e.g., woody biomass,^3^ or that quantify only some of the biomass components, e.g., carbohydrates.^7^ However, when putting together the partial values reported, some numbers do not add up. Furthermore, biomass classification criteria also differ among the scientific community. In some cases, biomass is classified according to its origin,^8^^,^^9^ i.e., woody biomass, agricultural biomass, aquatic biomass, municipal solid waste (MSW), and sewage sludge. However, the way these origins are defined may also vary from source to source. In other cases, biomass is classified according to composition,^7^^,^^10^ i.e., polysaccharides, lignin, proteins, and lipids, but even in this classification the criteria slightly differ. The disparity of available data calls for an agreement among the scientific community and stakeholders involved on biomass and biobased materials, and AI tools should facilitate the development of standards by finding patterns in available data that would be hard to identify otherwise. Data-driven standards will enable a sound analysis of the current landscape and resources available toward biomass valorization and biorefinery technology.

Location, cultural, and social aspects are also key factors to consider. The so-called developed countries have systems for biomass management and valorization, whereas developing countries mostly burn it for heat generation.^11^ In some cases, biomass is commonly used as fuel for cooking by lower-income households and agrarian communities, which amounts to the 38% of the world population. The CO_2_ emissions derived from this cooking, in addition to the combustion of crop residues in agriculture, scales up to nearly 18% of total CO_2_ global emissions.^3^ More technical challenges also exist, which range from logistic, handling, and storage costs^12^^,^^13^ to physical and chemical efficiency of processes and optimization, including extraction, catalysis, or depolymerization.^14^

Research, development, and investment is needed to achieve deeper understanding of composition-process-structure-property relationships in biomass carbon mining, and more importantly to scale up biorefinery processes that allow us to efficiently utilize renewable resources^15^ for the production of fit-for-purpose biobased materials. To assist with this composition-process-structure-property relationships, ML algorithms are being used to identify optimal hydrothermal processing (HTP) conditions,^16^^,^^17^ although the complexity of HTP reactions and the different partitioning of chemical species between solid and liquid phases during the process make mechanistic modeling extremely challenging.^18^ Despite the challenges ahead, the advantages of incorporating computational modeling into biomass-for-HTP or biobased-from-HTP materials, as well as the new opportunities arising from the integration of AI/ML into the material design and the biomass data analysis, are out of question. Thus, this perspective article focuses on the use of computational techniques, i.e., density functional theory (DFT) calculations, molecular dynamics (MD) simulations, coarse grained (CG) models, and AI/ML methods for the development of biobased materials obtained from HTP, including hydrothermal liquefaction (HTL) and hydrothermal carbonization (HTC), the two hydrothermal processes that use subcritical water. It also reviews the current landscape of applications arising from the production of these biobased materials in the context of a circular economy to highlight the current challenges and to anticipate potential avenues for development.

## Biomass: A key resource for the transition to a circular economy

### Challenges on classifying biomass origin and quantifying its availability

Biomass is usually classified following different criteria, which depend on biomass source, composition, or even the intended application of the feedstock. Following the classification of solid biofuels in the ISO 17225-1:2021^19^ and according to their origin, we have divided biomass into four groups: agricultural and horticultural biomass, wood and woody biomass, aquatic biomass, and animal and human waste biomass.

**Figure 2:**
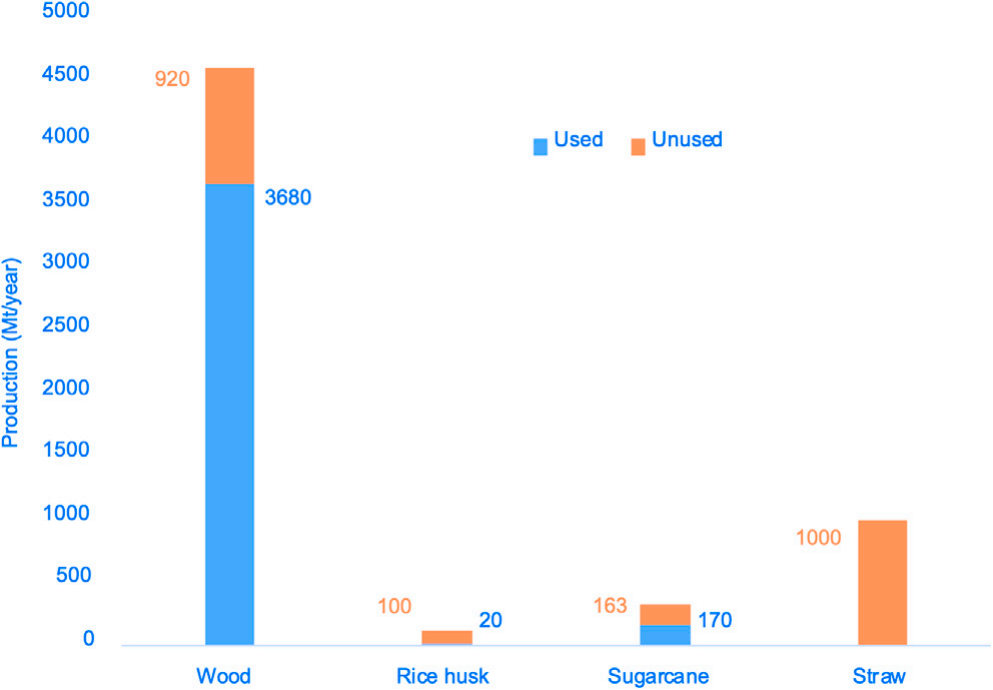
Yearly production of wood-derived and agricultural biomass worldwide in metric tons, according to different studies across 2011, 2012, and 2019: wood, rice husk, sugarcane bagasse, and straw.

In agriculture and horticulture, over 1,000 Mt of biomass coming from the processing of crops go to waste every year^7^ (see Figure 2). Some projections suggest that the land used for agriculture in developing countries will increase 13% by 2030.^3^ Burning this waste or leaving it untreated causes environmental issues,^20^ and it misses an opportunity for mining carbon from waste, promoting the circular economy in agriculture.

Similarly, it has been estimated that around 4,600 Mt of wood-derived biomass are produced every year. Worldwide, 60% is used for energy generation, 20% is used in industrial “round wood,” and the other 20% remains unused as production loss (see Figure 2).^3^ Second-generation biorefineries, which use wood-derived biomass that do not compete with food, are already addressing these issues, although they are still facing several challenges.^21^ For instance, during lumber production, 55% of the tree is still lost as waste, while it is estimated that 51% of this waste is recoverable^22^ for biofuel and biobased materials production. Figure 2 summarizes the production (Mt/year) of wood,^3^ rice husk,^20^ sugarcane bagasse,^8^ and straw.^3^

**Figure 3:**
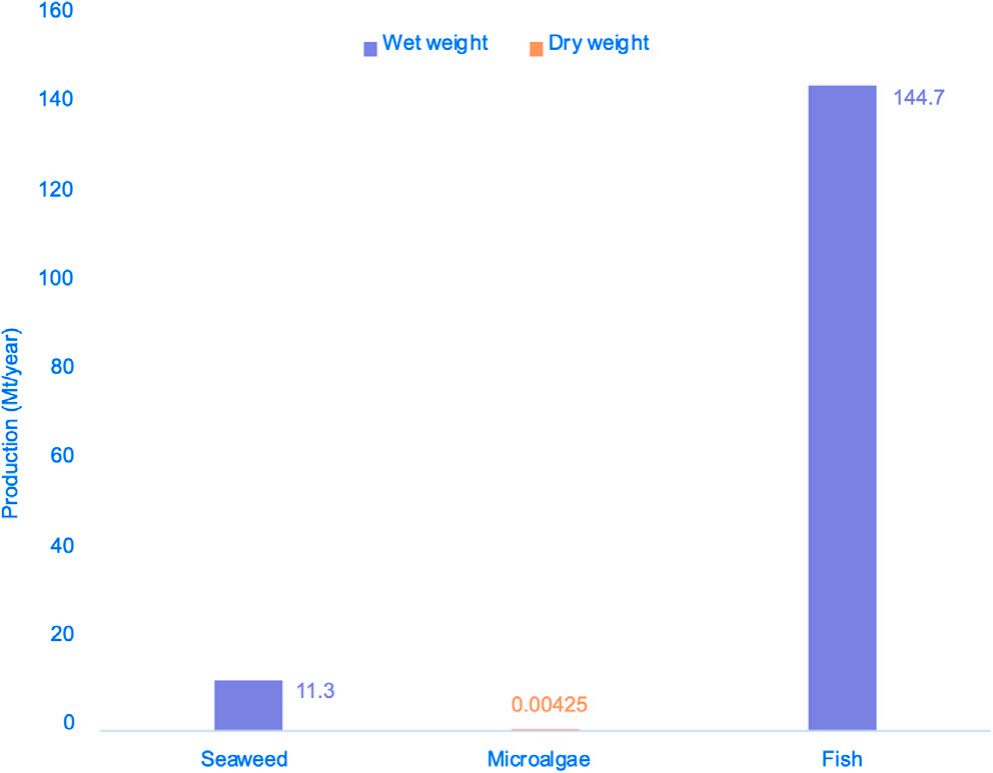
Yearly production of aquatic biomass worldwide in metric tons, according to different studies in 2010, and 2011: seaweed, microalgae, and fish.

In the case of aquatic biomass production (see Figure 3), the estimated cultivation of 4,250 dry weight tons of microalgae^23^ seems incongruent with the 11.3 million wet tons of seaweed produced yearly.^24^ Also, it is not clear whether the 92 Mt of fish from capture fisheries and the 52.7 Mt from marine and freshwater aquaculture reported yearly worldwide^25^ should be included in aquatic or animal biomass. This points out the challenges already mentioned in biomass quantification. Figure 3 summarizes the production (Mt/year) of seaweed,^24^ microalgae,^26^ and fish.^25^

**Figure 4:**
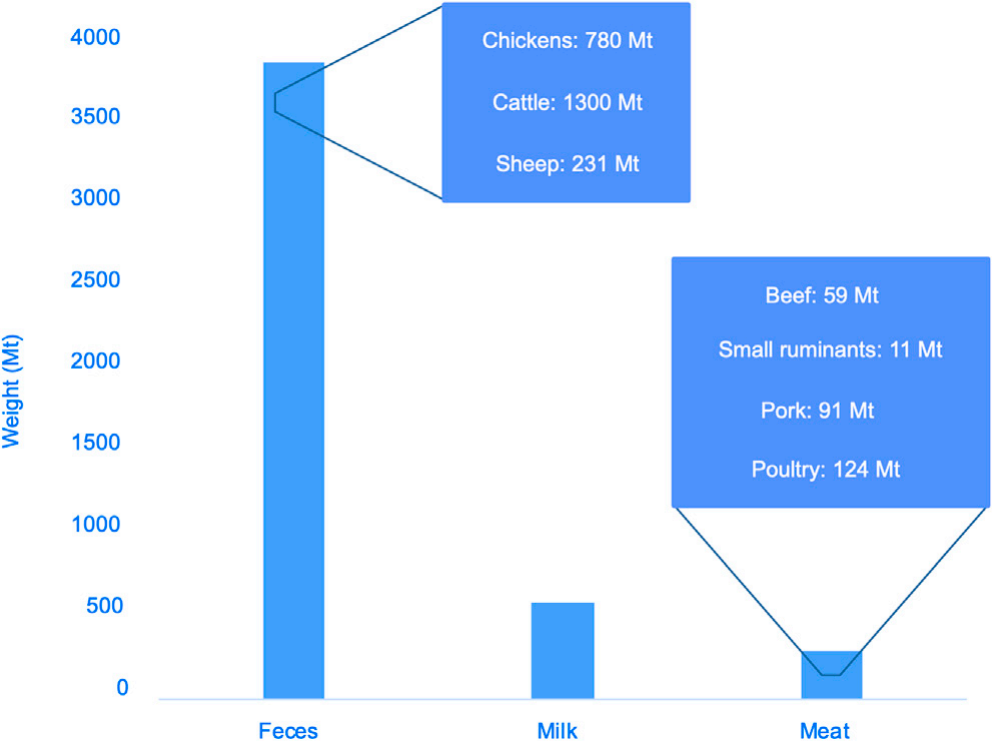
Yearly production of animal biomass worldwide in metric tons, according to different studies across 2010, 2011, 2012, 2018, and 2019: feces, milk, and meat.

Similar challenges exist with the classification of human and animal biomass waste. Sometimes, only feces are included in this category, the global production of which is estimated to be 3,900 Mt (i.e., 780 Mt come from chickens, 1,300 Mt come from cattle, and 231 Mt come from sheep),^24^ while in other studies, MSW, which contains food waste and furniture, is included as human waste, too. Further issues arise from quantifying animal or livestock waste since it does not only include manure but meat and bones (see Figure 4). Similarly, feathers from poultry are also biomass, but not usually included. Figure 4 summarizes the production (Mt/year) of feces,^24^ milk,^27^ and meat.^24^

Despite the inconsistencies in some data, and the challenges in quantifying and classifying biomass production and waste, the large magnitude of these resources is unquestionable. The total production of biomass waste from all these different sources and the large percentage that is currently underutilized reinforces the potential to transition from a petroleum-based to a biobased economy. However, more research, technology development, policy, and impact investment must be devoted to move from traditional mining of fossil carbon to waste carbon mining. Moreover, AI and ML are expected to contribute to data analysis and classification. For instance, combining techniques such as unmanned aerial vehicle remote sensing inversion and ML^28^^,^^29^ is one of the avenues to explore for tackling these challenges in the future.

### Mining biomass building blocks for targeted applications

**Figure 5:**
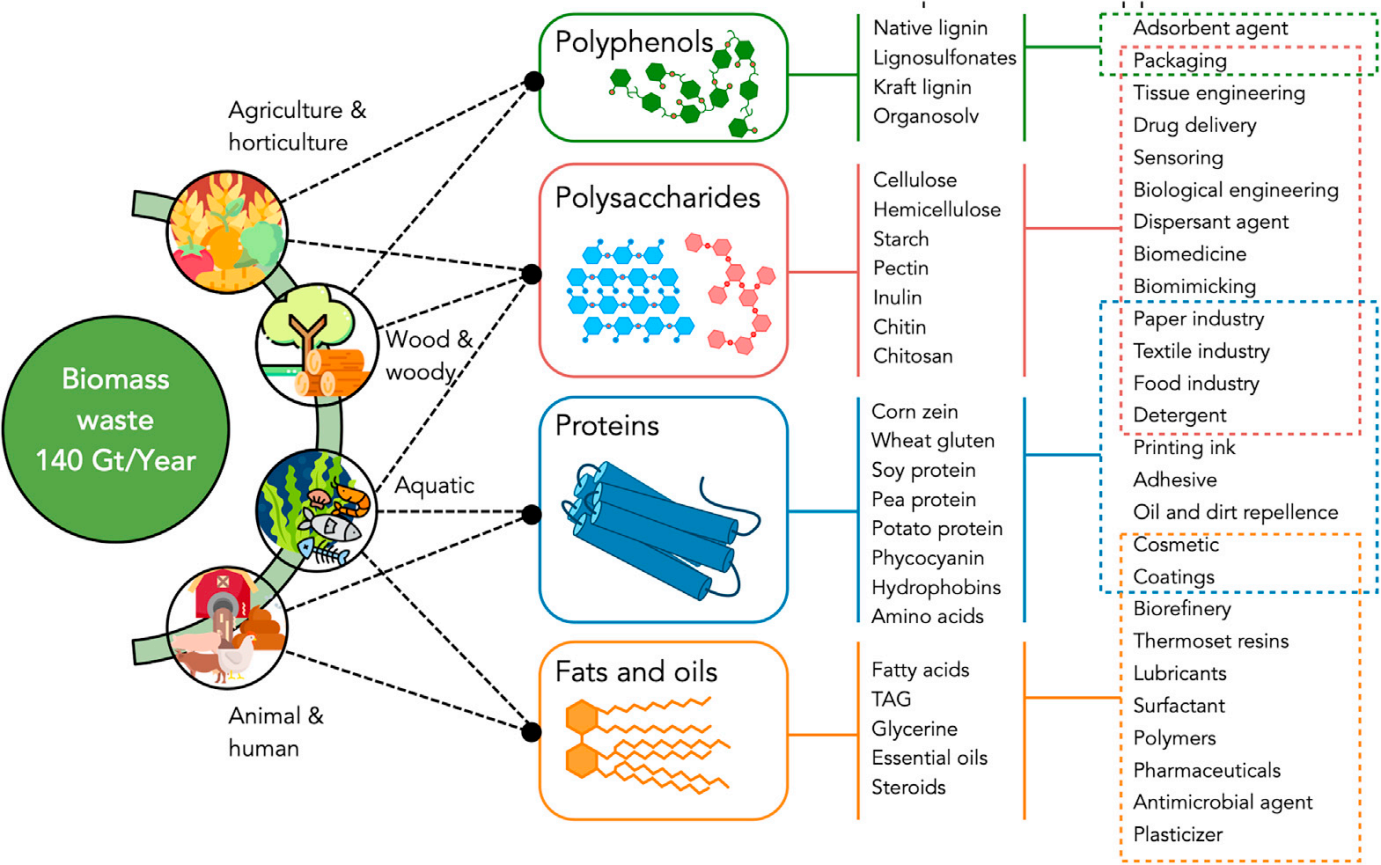
Classification of biomass waste by origin with the different building blocks and chemical components available in each case The current applications of the chemical components are shown on the right-hand side of the figure, as discussed in the text.

Feedstock’s chemical features determine the properties, the possible transformations to undergo,^30^ and, ultimately, the applications of biomass and biobased materials. The main components of biomass are polysaccharides, polyphenols (i.e., lignin), proteins, fats and oils,^7^ as well as some bioactive molecules, such as antioxidants, flavonoids, lignans, and carotenoids,^31^ which can be also isolated for utilization (see Figure 5).

Approximately, 75% of the worldwide production of biomass is composed of polysaccharides and lignin.^32^ Lignin itself comprises 25%–35% of the woody biomass,^33^ and it is the main natural source of biobased aromatic compounds.^34^ The main polysaccharides of plant origin include cellulose, hemicellulose, starch, inulin, and pectin, whereas those of animal and fungal origin include chitin and chitosan.^34^

However, despite the knowledge on biomass composition, there are several challenges ahead to separate biomass components efficiently, depolymerize them into fundamental building blocks, and either extract biobased platform chemicals or produce biobased materials from them. Thus, more control over biobased material production processes is needed. In fact, depolymerization processes is one area in which computational modeling and simulations are expected to help, both in understanding the depolymerization mechanisms and in designing better catalysts to produce high-value chemicals. Especially challenging for the success of biorefineries is the selectivity toward specific products^35^ and the efficient depolymerization of lignin.^36^ Lignin has been successfully applied in some applications,^37^ and its potential as a biological source of benzene, toluene, ethylbenzene, and xylene (BTEX) is unparalleled. There are many studies detailing the production of these chemicals from lignin on a small scale^38^^,^^39^ with current areas of focus being sustainability of production^40^ and selectivity of the process.^41^ Conceptual designs of lignin depolymerization processes in biorefineries have been investigated,^42^ and a range of routes to the production of renewable benzene have been discussed.^43^

Nevertheless, lignocellulosic biomass is generally being incorporated into a circular economy model thanks to biorefineries, with biochemical,^44^^,^^45^ mechanical,^44^ or thermochemical treatments. However, despite the wide range of processes available at the laboratory scale, challenges persist due to the intrinsic complexity of biorefinery technologies to process extremely wide and heterogeneous feedstock.

Some biobased materials are being produced in pilot plants and have found technical applications. For example, cellulose in different forms (e.g., nanocrystalline cellulose, nanofibrillated cellulose, bacterial cellulose) can be applied in fields such as tissue engineering,^46^ drug delivery,^47^ sensors,^48^ biomimicry,^49^ and even in polymer nanocomposites, replacing petroleum-based fillers.^50^^,^^51^ Starch, inulin, and pectin are used as a food additive and animal feed, and also applied in biodegradable packaging,^52^^,^^53^ or in detergent formulations to replace polyacrylates.^34^ Others like chitin and chitosan are useful in biomedical applications,^54^ and for treating industrial pollutants.^55^^,^^56^^,^^57^

In the case of fats and oils from biomass, with a global annual production of around 206 Mt (87% of vegetable origin and 13% of animal origin),^58^ similar applications to those from their petroleum counterparts have been achieved.^30^ Examples include the use of waste of vegetable oils and fatty acids, such as glycerol, citric acid, and cardanol, to produce renewable plasticizers,^59^ lubricants, surfactants, coatings, and precursors for producing biodiesel, polymers, pharmaceutical compounds, and cosmetics.^30^ Other examples include thermoset resins and antimicrobial agents.^60^ Meanwhile, protein-containing waste is used for animal feed, although it could be also a valuable source for manufacturing biobased materials^7^ like printing inks, grease-proof paper, coatings, adhesives, cosmetics, detergents, and plastics.^61^ However, valorizing protein-containing biomass usually results in a fuel-vs-food debate, which may be solved by separating essential and nonessential amino acids. For instance, feathers from poultry slaughterhouses have a protein content of 75% w/w^62^ of which 65% are nonessential amino acids to be used in non-food applications.

Despite the challenges associated with it, industrial application and utilization of biomass is growing, with an increasing number of medium-sized enterprises and startup companies employing technologies to produce high-value chemicals and alternatives to petroleum-derived materials from biomass. Table 1 highlights some startups and medium-sized enterprises that are successfully valorizing waste, e.g., agricultural wastes, industrial waste, end of life tires, cardboard, municipal waste, and exhausts from industrial processes, into biobased materials toward a more circular economy. One of the largest areas of interest in biobased material production are alternatives to petroleum-derived plastics by utilizing a range of biobased polymers (polyhydroxyalkanoates,^63^ polylactic acid,^64^ polyhydroxybutyrate,^65^ and polyethylene furanoate).

**Table 1:** Some start-ups and medium-sized enterprises currently valorizing waste biomass.

Company	Feedstock	Product	Application	Reference
ADBioplastics	Corn, sugarcane, and sugar beets	Bioplastics (polylactic acid)	Alternative to oil-based plastics	Bar-On et al.^66^
Aeropowder	Surplus feathers, cellulose	Thermal insulator (composed of feathers, cellulose film, and a biobinder)	Insulation; focused on alternatives to polystyrene packaging	Aeropowder^67^
Algaeing	Algae	Fibers and dyes	Biodegradable fibers and dyes for use in the textile industry	Algaeing et al.^68^
Amyris	Sustainably sourced sugarcane	Chemicals (squalene, cannabigerol, etc.)	Cosmetics, pharmaceuticals, food additives	Amyris^69^
Avantium	Plant-based sugars, non-food biomass	Bioplastics (polyethylene furanoate), industrial sugars, and lignin	Alternative to oil-based plastics. Alternative in chemical and material industries to fossil-fuel derived materials	Avantium^70^
Bio-bean	Waste coffee grounds	Chemicals (natural pyrazines), biofuels	Natural flavors from pyrazines, wood fuel alternative	Bio-bean^71^
Biocomposites Group	Natural fibers—flax, hemp, and jute	Terrafibre (biopolymer)	Erosion control blankets, consumer goods (as plastic alternative), automotive parts	Biocomposites Group^72^
BIohm	Mycelium	Construction material (mycelium panels)	Alternative to traditional building materials; focused on thermal and acoustic insulation	Biohm^73^
Biome	Sugarcane, potato starch, and cellulose (from trees and straw)	Bioplastics	Alternative to oil-based plastics	Biome^74^
Bioplastech Ltd.	Waste plastic (polyethylene, polyethylene terephthalate and polystyrene) treated with synthetic microbes	Bioplastics (polyhydroxyalkanoates)	Alternative to oil-based plastics: focused on adhesive and biosurfactant	Bioplastech Ltd.^75^
Biosynthetic technologies	Vegetable oils	Chemicals (organic fatty acids)	Alternative to oil-based lubricants in a range of fields (industrial, marine, automotive). Pharmaceuticals and cosmetic/personal care products	Biosynthetic technologies^76^
Black Bear Carbon B.V.	End of life tires	Carbon black	Coatings, inks, plastics, rubbers, tires	Black Bear carbon^77^
Bluecat paper	Food and food processing waste (coffee husk, banana fiber, corn husk, bagasse, tea waste)	Paper	Tree-free, feedstock high in cellulose (less chemical treatment required)	Bluecat paper^78^
Bolt Threads Inc.	Mycelium	Mycelium derived “leather”	Environmental fashion	Bolt Threads Inc.^79^
Calysta	Carbon dioxide	Glucose and proteins	More sustainable feedstocks for the aquaculture industry	Calysta^80^
Celluforce	Woody biomass	Cellulose nanocrystals	Polylactic acid additive (improving gas barrier properties), lubricants, particle suspension, latex additive, viscosity regulator in oil/gas	Celluforce^81^
Chaincraft	Organic waste and residues	Chemicals (medium-chain fatty acids)	Animal feed, lubricants, plasticizers, polymers, coatings, and flavors/fragrance	Chaincraft^82^
Clariant Ltd.	Agricultural waste (wheat straw, corn stover, sugarcane bagasse)	Biofuel (cellulosic ethanol production)	Alternative to oil-based fuel	Clariant Ltd.^83^
Cleanfiber Inc.	Cardboard	Cellulose	Insulation	Cleanfiber Inc.^84^
Eco-Shell	Waste shells from walnut processing	Walnut shell products (powders, granules, and pellets)	Blasting/polishing abrasives, exfoliant in cosmetics, water filtration	Eco-Shell^85^
ECOR Global	Waste materials (those they can source that have high lignin/cellulose content)	Construction material (similar material to MDF)	Alternative to traditional building materials, with the ability to be coated	ECOR Global^86^
Ecovative	Mycelium and hemp	Packaging, construction	Alternative to plastic and bioplastic film packing; focused on packaging customizability via manipulating growth. Can also be grown into products such as rafts and door cores	Ecovative^87^
Enerkem	Waste material (many types; textiles, non-recyclable plastics, wood residues, municipal waste, etc.)	Biofuels (ethanol, methanol)	Alternative to fossil-based fuels	Enerkem^88^
Envar	Biomass waste	Soil improver	Fertilizers	Envar^89^
Full Cycle Bioplastics	Food and organic waste	Bioplastics (PHA, polyhydroxyalkanoate)	Alternative to oil-based plastics	^90^
Gelatex	Gelatin, alginate, chitosan, zein, hyaluronic acid	Nanofibers (3D scaffolds and nanocarriers)	Cell-cultured meat, tissue engineering	Gelatex^91^
Genecisis	Food waste	Bioplastics (polyhydroxyalkanoates)	Alternative to oil-based plastics	Genecis^92^
Genomatica	Plant sugars	Plant-based nylon, butylene glycol, beta-hydroxybutyrate, bio-1,4-butanediol	Alternatives to oil-derived polyesters/polyamides, and plastics. Chemicals for cosmetics and nutrition	Genomatica^93^
Gingko Bioworks	Microorganisms	Microorganisms (developed with synthetic biology) for waste treatment and a variety of other applications	Waste treatment, vaccine supply chain support, “living” medicines	Ginkgo Bioworks^94^
Global Bioenergies	Sugarcane, beets, wheat, corn, straw, and wood	Chemicals (isobutene)	Fragrances, tires, biofuels, waterproofing	Global Bioenergies^95^
Greenhope	Cassava	Bioplastic	Alternative to oil-based plastics	Greenhope^96^
InventWood	Sustainably sourced cellulose-rich materials	Construction materials	Alternative to wood and aluminum; cladding/facades, automotive parts, shipping containers, furniture, industrial equipment	Invent Wood^97^
Jiva Materials	Flax and other fibrous biomass	Biodegradable and recyclable PCBs (printed circuit boards)	An alternative to circuit boards made from glass fibers	Jiva Materials^98^
Lactips	Casein	Bioplastic (polylactic acid)	Alternative to oil-based plastics; focused on solubility in cold water and compostability	Lactips^99^
LanzaTech	Industrial emissions (carbon recycling)	Chemicals (fuels and plastic precursors)	Alternative to traditional oil-based plastics and fuels	LanzaTech^100^
LOLIWARE Inc.	Seaweed	Bioplastic (pellets derived from seaweed)	Alternative to oil-based plastics; focusing on biodegradable straws. Previously manufactured edible straws and cups	^101^
Mango materials	Methane	Bioplastics (polyhydroxyalkanoates)	Alternative to oil-based plastics	^102^
Marinatex	Waste from fishing and shellfish industries	Bioplastics	Alternative to oil-based plastics; focused on home composting	LanzaTech^103^
MYCEL	Mycelium	Mycelium-derived “leather”	Environmental fashion	MYCELL^104^
Naifactory Lab	Waste olive pits	Construction material (like wood but can be poured and molded), a clear variant of the material is also available	Home furnishings such as lamps and chairs	Naifactory Lab^105^
NatureWorks	Lactic acid derived from fermented sugars of sugarcane, sugar beets, and cassava	Bioplastics (polylactic acid)	Alternative to oil-based plastics; focused on the food packaging industry and 3D printing filaments	NatureWorks^106^
Newlight Technologies Inc.	Carbon dioxide	Bioplastics (polyhydroxybutyrate)	Alternative to oil-based plastics; focused on circumventing traditional feedstocks (oil or plant matter)	Newlight Technologies Inc.^107^
Notpla	Seaweed	Bioplastics	Alternative to oil-based plastics; focused on novel and innovative applications	Notpla^108^
Novamont	Vegetable oils	Matrol-BI (Bio lubricant, 100% biodegradable)	Machinery operating within areas of ecological sensitivity. (Hydraulic fluids, greases, transmission fluid, dielectric fluids)	Novamont^109^
Origin Materials	Sustainably harvested wood, agricultural waste, wood waste, and cardboard	Bioplastics (PET), chemicals (chloromethyl furfural, levulinic acid, furfural)	An alternative to oil-based plastic. Platform chemicals	Origin Materials^110^
PENNSACO TECHNOLOGIES	Biomass waste	Carbon-negative hydrogen fuel and biochar	Alternative fuel source. Regenerative agriculture and carbon sequestration	PENNSACO TECHNOLOGIES^111^
Phase Change Solutions	Plant-based feedstocks	Phase-change materials	Biobased materials for heating/cooling	Phase Change Solutions^112^
Phool	Waste from floral temple offerings	Compost, oils	Fertilizer, incense sticks, essential oils	Phool^113^
Plantic	Corn starch	Bioplastics	Alternative to oil-based plastics; focused on the food packaging industry	Plantic^114^
Pro Farm	Microbes, plant extracts, fatty acids, and pheromones	Biopesticides	Low-impact pesticides (reducing exported harmful residues and reducing health risks to workers)	Marrone Bio Innovations Inc.^115^
Seramic Materials	Industrial solid waste	Ceramics	Alternative to conventional ceramic materials	Seramic Materials^116^
Skipping Rocks Lab	Seaweed	Biopackaging (sodium alginate and calcium chloride)	Alternative to oil-based plastics; focused on edible sachets and water bottles	Skipping Rocks Lab^117^
Solugen	Agricultural waste materials (i.e., sugarcane and corn) with AI developed catalysts and enzymes	Chemicals (hydrogen peroxide, chemicals for ion detection in water)	Alternative to chemical production processes that typically use petroleum-based feedstocks	Solugen^118^
Solutum	Biomass	Bioplastic	Alternative to oil-based plastics	Solutum^119^
Sulapac	Sustainably sourced crops, waste wood from industry	Bioplastics	Alternative to oil-based plastics and microplastics	Sulapac^120^
Svevia	Lignin	Asphalt biobinder	An alternative/replacement for bitumen in asphalt	Svevia^121^
Sweetwater Energy	Sugars from natural fibers (i.e., wood chips/straw)	Cellulosic sugars, Lignin	Fermentation of sugar into chemicals (biofuels, lactic acid, succinic acid). Production of other chemicals from lignin (phenols, vanillin)	Sweetwater Energy^122^
T I P A	Corn starch, sugarcane	Bioplastics	Alternative to oil-based plastics; focused on packaging that you can compost at home	TIPA^123^
Timeplast	Plastic resins and bioplastics. A treatment process makes these soluble	High-molecular-weight, soluble, bioplastics	An alternative to traditional oil-based plastics; focused on water solubility	Timeplast^124^
UBQ	Unsorted municipal waste (typically household waste containing many organic materials)	Bioplastics	Alternative to oil-based plastic; compatible with injection molding and 3D printing	UBQ et al.^125^
Utopia Plastix	Low-maintenance crops	Plant-based compounded resin that can be used in injection molds	Alternative to oil-based plastics; cutlery, straws, films, etc.	Utopia Plastix et al.^126^
Visolis	N/A	Data analytics platform for synthetic biology and catalysis development	Promising molecular candidates for biobased alternatives	Visolis^127^
Woodoo	Wood, plant resins	Augmented wood	Alternative to traditional building materials such as steel and concrete	Woodoo et al.^128^
Xampla	Plant material (peas)	Bioplastics (from plant protein)	Alternative to oil-based plastics; focused on soluble films	Xampla^129^

Comprehensive studies should be carried out to discern the potential side effects and unwanted consequences of these new business models, as logistical, processing, and end-of-life treatments might increase the impact on our environment, not to mention food-competitive crops production. Bioplastics derived from underutilized waste streams that can be home composted seem the most promising alternative to petroleum-derived plastics, and therefore it is important to avoid materials that can only be composted industrially, taking years to fully breakdown outside of these facilities.

In addition, there are numerous projects being funded in partnership by the European Union and the biobased industries consortium to develop new technologies that process and valorize waste biomass, including dairy processing by-products, mushroom farm by-products (offcuts and soil), olive leaves, and biorefinery residual sugars.^130^

## Biomass treatment to produce biobased materials

Biomass naturally occurring molecules can be extracted and utilized, while biomass carbon can be further valorized by decomposing and repolymerizing biomass into biobased materials,^131^ either by biological methods^25^ like fermentation and digestion or by thermochemical ones, such as torrefaction, pyrolysis, or HTP. The yield of the processes, and the structure of the materials produced thereof, depends on the biomass composition, temperature, heating rate,^132^ and water content,^133^ among other factors. Computational tools are expected to assist in understanding transformation mechanisms of biomass into biobased molecules and materials, which will eventually lead to improvements in the processing methods and further control of the products obtained. However, integrating these computational tools into material design procedures and process engineering is challenging due to intrinsic limitations of the models and the computational capabilities.

### Biological processes

Biological processes include fermentation and anaerobic digestion. Fermentation is a well-established alternative for producing chemicals that replace petroleum derivatives. However, its scaled-up application is still rather limited due to difficulties in the product recovery and removal of by-products that are toxic to microorganisms at certain concentrations, which affect the yield.^30^^,^^134^ Anaerobic digestion is mainly used for the production of methane and fertilizer recovery.^135^^,^^136^ A detailed discussion on biological process goes beyond the scope of this work.

### Thermochemical processes

Within thermochemical processes, direct combustion is a common practice with agricultural waste, especially, in developing countries,^6^^,^^137^ while torrefaction and pyrolysis are more widely used across industries and locations to decompose organic matter under a dry and inert atmosphere, i.e., water evaporation and absence of oxygen are required. They only differ in the operating temperature: torrefaction takes place at temperatures of 200°C – 350°C,^133^ while pyrolysis uses temperatures in the range of 300°C – 800°C.

**Figure 6:**
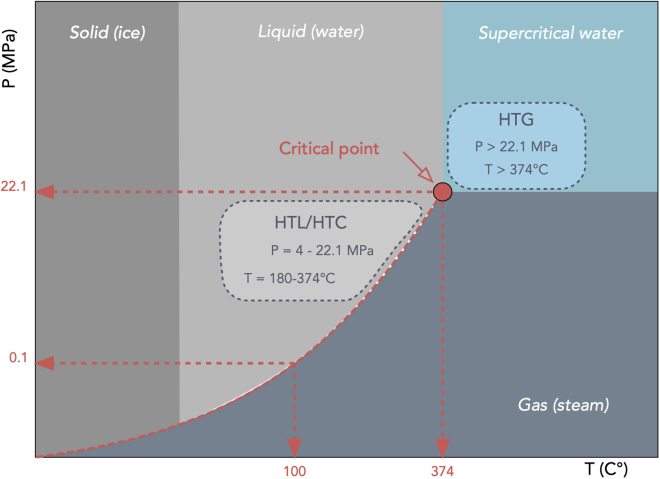
Phase diagram for water, highlighting the conditions at which HTL, HTC, and HTG take place in relation to water’s critical point. The diagram also indicates the areas in which water is solid, liquid, gas, or supercritical water, depending on the pressure (MPa) and the temperature (°C) conditions.

Differently, HTP does not require dry conditions but uses subcritical water as a reactant, solvent, and/or catalyst. This is an advantage when processing wet biomass. Depending on the conditions of the process, HTP is classified into HTL or HTC, although hydrothermal gasification (HTG) is also possible with supercritical water^138^ (see Figure 6), where H_2_, CO_2_, and CH_4_ are formed.^139^^,^^140^

Three main different fractions are obtained from the HTP of biomass, i.e., hydrochar, biocrude oil, and an aqueous phase, although some gases are also formed.^141^^,^^142^^,^^143^^,^^144^ Hydrochar is a carbon-rich solid material, while biocrude oil is a black, thickened bitumen-like fluid. Both hydrochar and biocrude oil, as well as the aqueous phase and the gases, can be valorized, intrinsically contributing to the development of more circular processes. In general, HTL predominantly produces biocrude oil,^145^ while HTC produces a larger fraction of a solid hydrochar. The yield, the type of reactions, and therefore the composition of the products depend on the raw materials, temperature, pressure, reaction time, size of biomass particles, catalysts, and reaction medium.^137^^,^^146^ Computational tools are expected to help optimize all these variables in hydrochar and biocrude production, although its contribution is still in the early stages.

Valorizing the aqueous phase is also needed to achieve a zero-waste HTP processes, and to reduce the energy loss and extra costs associated with waste water treatment. In fact, it has been reported that up to 40% of the carbon in the feedstock can be found in the aqueous phase after conversion.^147^ Suitable solutions are the extraction of valuable organic molecules in this aqueous phase, their conversion into gas fuels such as methane or hydrogen by means of catalytic processes and HTG,^148^^,^^149^ or the recirculation of water as reaction medium in the hydrothermal process itself. The last option increases the concentration of nitrogen and some heavy metals in the final biocrude oils and hydrochar,^150^ which implies an increase of pollution, if used as biofuels,^151^ but improves biobased materials performance in some applications like supercapacitors,^152^ redox flow batteries,^153^ catalysis, and contaminant adsorption.^152^^,^^154^ In fact, understanding the role of functional groups such as pyridinic, pyrrolic, and quaternary nitrogen^155^^,^^156^ during processing, as well as in the biobased materials produced via HTP, is critical to unlock new technological avenues. This is where computational modeling and AI come into play to analyze large datasets and to identify the factors that control HTP reaction mechanisms toward the formation of nitrogen-rich biobased materials.

## Development of biobased computational materials

The use of computational simulations, AI, and ML for the development of tailored biobased materials that mimic Nature’s efficiency and circularity faces the intrinsic challenge of closing the gap between the scale of process engineering and the nanoscale at which chemistry operates. On the processing side, depending on the specific treatment, processing parameters, and type of biomass, the yields and composition of hydrochar, biocrude, and aqueous phase vary. On the modeling side, *ab initio* quantum mechanical methods, DFT, MD simulations, and CG models provide understanding of structure-property relationships, solvent effects, degradation mechanisms, thermal conversion, mechanical performance, adsorption, and corrosion mechanisms of biomass and biobased materials, but their applicability is sometimes limited. In the following sections, Tables 2, 3, 4, and 5 summarize HTP experiments of different types of biomasses and the resulting compositions of the main fractions obtained. These experimental results are discussed against some available computational studies, pointing out pending challenges and potential future directions in the context of a circular economy.

**Table 2:** Chemical functional groups found in chars from HTC at different processing conditions of different biomass model compounds, together with the reference where this characterization was reported.

Biomass type		HTC Conditions	Hydrochar’s functional groups	Reference
Polysaccharides	Cellulose	200-250°C, 2-4 h	Aliphatic, aromatic, carbonyl, hydroxyl, carboxylic groups, esters, lactones—oxygen in the core forms stable groups (i.e., ether, quinone, pyrone), whereas the oxygen functionalities in the outer layer consist of more reactive/hydrophilic groups (i.e., hydroxyl, carbonyl, carboxylic, ester)	Sevilla and Fuertes^157^
	Cellulose	225-265°C, 20 h	Hydroxyl, aliphatic, aromatic, carboxyl/carbonyl groups	Kang et al.^158^
	D-xylose	225-265°C, 20 h	Hydroxyl, aliphatic, aromatic, carboxyl/carbonyl groups	Kang et al.^158^
	α-D-glucose, D-(+)-sucrose, and starch	170-240°C, 0.5–15 h	Carbonyl, quinone, ester, or carboxyl; hydroxyl, ester, or ether; aromatic, aliphatic; hydroxyl or carboxyl	Sevilla et al.^159^
	D(+)-glucose, D(+)-xylose, maltose, sucrose, amylopectin, starch	180°C, 24 h	Ketones, aldehydes, carboxylic acids; aliphatic and ether groups	Titirici et al.^160^
Lignin		225-265°C, 20 h	Hydroxyl, aliphatic, aromatic, carboxyl/carbonyl groups (polyaromatic and polyphenolic hydrochar)	Kang et al.^158^
Fatty acids and lipids			No characterization available	
Proteins			No characterization available

**Table 3:** Molecules and/or chemical functional groups identified in biocrude oils from HTL at different processing conditions of different biomass model compounds, together with the reference where this characterization was reported.

Biomass type		HTL conditions	Elemental composition of biocrude oil	Reference
Polysaccharides	Starch	Isothermal	Ketones, phenols, indanone derivatives, furan derivatives, benzene derivatives	Gollakota and Savage^161^^,^^162^
		Fast	Ketones, phenol, furan derivatives, benzene derivatives, acids	
	Cellulose	Isothermal	Ketones, phenols, indanone derivatives, furan derivatives, benzene derivatives	
		Fast	Phenols, furan derivatives, benzene derivatives, acids, alcohols, esters, hydrocarbons	
	Amylopectin	Isothermal	Ketones, phenols, indanone derivatives, furan derivatives, benzene derivatives	
		Fast	Ketones, phenols, furan derivatives, acids, alcohols, esters, hydrocarbons	
	Amylose	Isothermal	Ketones, phenols, indanone derivatives, furan derivatives	
		Fast	Ketones, phenols, furan derivatives, benzene derivatives, acids, alcohols, esters, hydrocarbons	
	Pectin	Isothermal	Ketones, phenols, indanone derivatives, furan derivatives, benzene derivatives	
		Fast	Ketones, phenols, furan derivatives, benzene derivatives	
	Chitin	Isothermal	Ketones, phenols, indanone derivatives, benzene derivatives, nitrogenous compounds	
		Fast	Ketones, phenols, furan derivatives, hydrocarbons, nitrogenous compounds	
Lignin		Light oil, isothermal, NaOH 2%	Phenol, m/p-cresol, guaiacol, creosol, 3-methoxycatechol, p-ethyl-guaiacol, syringol, 4-propylguaiacol	Ciuffi et al.^163^
		Light oil, isothermal, NH_4_OH 8%	Phenol, m/p-cresol, guaiacol, creosol, catechol, p-ethyl-guaiacol, 3-methylcatechol, syringol, 4-ethylcatechol	Ciuffi et al.^163^
		Fast	Catechols, anisoles, alkyl phenols, guaiacols, benzene derivatives, others (xanthene, 2,3,4-trimethoxybenzoic acid, 2-phenyl-4-tert-butylphenol, coniferaldehyde, 1,2-diphenylethanone, guaiacylacetone)	Yong et al.^143^
		Isothermal, additives	Catechols, phenol, anisoles, alkyl phenols, guaiacols, phenolic dimers, other compounds (o-acetylphenol, 1,2-dimethoxybenzene, xanthene, 2-benzyl-4-methoxyphenol, retene, diethyl oxalate)	Belkheiri et al.^164^
Fatty acids and lipids		Isothermal, potassium phosphates	Acids/esters, alcohols	Ding et al.^165^
		Isothermal	Hexadecenoic acid, oleic acid, 4-octadecanolide	Teri et al.^144^
Proteins		Isothermal, potassium phosphates	Nitrogenous compounds, phenols, ketones/aldehydes, hydrocarbons, acids/esters	Ding et al.^165^
			Phenol, 2-pyrrolidine, piperidine, indole, hexadecanamide, oleamide	Teri et al.^144^

**Table 4:** Molecules identified in the aqueous phase from HTC at different processing conditions of different biomass model compounds, together with the references where this characterization was reported.

Biomass type		HTC Conditions	Molecules in the aqueous phase	Reference
Polysaccharides	Chitosan	180°C, 12 h	Amino-functionalized fluorescent carbon nanoparticles	Yang et al.^166^
	Glucose, sucrose, starch	170-240°C, 0.5–15 h	Furfural, hydroxymethyl furfural, acids, and aldehydes	Sevilla and Fuertes^159^
	D(+)-glucose, D(+)-xylose, maltose, sucrose, amylopectin, starch	180°C, 24 h	HMF, furfural, 4-oxo-pentanoic acid	Titirici^160^
	Cellulose and hemicellulose	140-240°C, 0.5–24 h	Furfural, 5-HMF	Borrero-López^167^
Lignin		140-240°C, 0.5–24 h	Vanillin, guaiacyl acetone, syringaldehyde, syringol, acetosyringone, guaiacol, phenol, acetovanillone, creosol	Borrero-López^167^
Fatty acids and lipids			No information available	
Proteins			No information available

**Table 5:** Molecules identified in the aqueous phase from HTL at different processing conditions of different biomass model compounds, together with the references where this characterization was reported.

Biomass type	HTL Conditions	Molecules in the aqueous phase	Reference
Polysaccharides	Isothermal	Cyclic ethers, alcohols, acids	Yang et al.^168^
Lignin	Isothermal, additives	Catechols, phenol, anisoles, alkyl phenols, guaiacols, phenolic dimers, other compounds (1,2-dimethoxybenzene, salicylic acid, ethanedioic acid, diethyl ester)	Belkheiri et al.^164^
Fatty acids and lipids	Isothermal, K_2_CO_3_	Carboxylic acids, fatty acids, dicarboxylic acids, oxygenated aromatics, cyclic oxygenates, nitrogenates	Madsen et al.^169^
Proteins	Isothermal, K_2_CO_3_	Carboxylic acids, fatty acids, dicarboxylic acids, oxygenated aromatics, cyclic oxygenates, nitrogenates	Madsen et al.^169^
	Isothermal	Nitrogenous compounds	Yang et al.^168^

Models are limited in size, and complexity, but still able to provide valuable insights. Plant-derived biomass components, especially cellulose, hemicellulose, and lignin, as well as the interactions between the three,^170^ have been probably the most studied materials to date. DFT studies are usually limited to very small molecular systems and focused on polymerization and depolymerization mechanisms. For example, DFT methods helped to elucidate the initial steps of lignin polymerization,^171^ the conversion of xylose (the main component of hemicellulose) to furfural,^172^ or the development of biobased steel corrosion inhibitors.^173^

At scales beyond those achievable by DFT calculations, MD simulations are used to accurately describe supramolecular assemblies. Examples include studies on the relation between lignin sequences and its 3D structure,^174^ the dissolution and aggregation mechanisms during lignin’s enzymatic hydrolysis,^175^ or the mechanisms of biomass degradation in THF and water.^5^ Some force fields like CHARMM have been parametrized to accurately describe lignin-lignin, lignin-water, and lignin-hemicellulose interactions^176^ and are helping with understanding the pyrolytic degradation of native cellulose.^177^ MD simulations have also been employed to study the role of water in the HTP of lignin^178^ and the depolymerization mechanisms that occur during HTL of cellulose, hemicellulose, lignin, and lipids, leading to further understanding of how biomass breaks down during HTL, and to the development of a biocrude oil yield prediction model.^179^ Other advancements have also been made with the addition of external forces to the MD simulation, mimicking agitation of the mixture and providing insights into the dissolution of cellulose into individual chains.^180^ MD simulations have also been used to study alternatives to petroleum-derived plastics. For example, PLA/PHB (polylactic acid/polyhydroxybutyrate) mixtures were investigated to study their miscibility and properties of polymer blends.^181^ In the future, new pair potential that learn from data using differentiable simulations, as developed by Wang et al.,^182^ are expected to further increase the accuracy and efficiency of these simulations, and to optimize multiple biomass and biobased systems simultaneously.

At even larger scale, CG models have been developed to perform simulations closer to the mesoscale, which is the theoretical barrier for atomistic simulations. In particular, the MARTINI force field^183^ has been proved to be the most applicable CG force field, with applications in several areas, e.g., PLA to develop microcapsules for drug delivery.^184^ However, when coarse graining, selected atoms are grouped into pseudo-beads to drastically accelerate the simulations. Such CG procedure induces information losses, which makes restoring fine-grained (FG) coordinates from CG coordinates a long-standing challenge. Wang et al.^185^ have been able to encode the FG uncertainties into an invariant latent space and decode them back via equivariant convolutions. This is an outstanding achievement that opens an avenue for atomistic simulations at the mesoscale, by sequentially CG and FG materials systems. Using this approach for biomass and biobased materials is a pending task. Nevertheless, more traditional CG models have been developed for cellulose nanocrystals bundles,^186^^,^^187^ and natural cellulose fibrils,^188^ and recent studies have been carried out on the conformational changes in cellulose microfibrils.^189^ Given that the dissolution process of cellulose involves charged species and ionic liquids, polarizable CG models for solvents other than water should be developed in the future.

Another aspect in which MD simulations and CG models have been proved very useful is the description of interactions between biomass materials and other biomaterials like silk fibroin and chitosan^46^ for biomedical applications. Furthermore, not only the individual components but also wood has been studied using MD simulations. Following an early study with a CG model of wood cell walls,^190^ a molecular model of the wood cell wall material with atomistic resolution was used to assess the mechanical behavior under shear loading at the molecular level.^191^ CG and FG modeling, as developed by Wang et al.,^185^ should also be used for wood-focused simulations.

### Designer hydrochars

Depending on the degree of dehydration, the chars obtained from HTP can be classified as primary and secondary chars. For simplicity, we will refer to any char from HTP as hydrochars. Predominantly produced from HTC, hydrochars possess high energy and mass density and a porous structure that can be computationally designed. The physicochemical properties of the chars vary depending on the type of biomass, the specific processing conditions, the use of catalysts, and the application of any activation procedures.^141^ Hydrochars are challenging to characterize, which is hindering the development of models at different length scales. Table 2 summarizes the functional groups found in hydrochars from HTC at different processing conditions of different model compounds. Most studies focus on cellulose and lignin, although HTC of other materials like spent sugar beets,^192^ or even some mixtures of components like glucose and egg white ovalbumin,^193^ has been performed.

In the case of lignin, lower temperatures and shorter processing times promote the formation of phenolic monomers and dimers, whereas at higher temperatures and longer times, demethoxylation and alkylation of phenolic compounds takes place. Hydrochar formation from lignin is enhanced at elevated temperatures, especially under supercritical conditions.^143^

General applications of HTC hydrochars include energy storage, electrocatalysis, heterogeneous catalysis, gas storage, water treatment, bioenergy, or catalytic conversions. More detailed information on these applications can be found in a very thorough review by Nicolae et al.^141^

HTL of biomass also produces hydrochar, although, to our knowledge, its characterization has been mostly limited to elemental analysis, with some exceptions for the case of lignin^194^ and lipids.^195^ This is probably due to the fact that the solid residue is mostly inorganic material.^196^

On the modeling side, very few DFT studies involving hydrochar have been reported, although the few models that have been developed for biochar should be easily transferable. Hydrochar synthesized from glucose and treated with hydroxide and carbonate salts of potassium and sodium was studied using DFT^197^ on a previously developed hydrochar model with furan rings.^198^ A second study developed a model of graphitized hydrochar to study the sorption of methyl orange and methylene blue on a Fe-doped porous graphite hydrochar derived from the HTC of dry cotton straw.^199^ DFT has also been used as an auxiliary method in characterizing both hydrochar and biochar,^200^ demonstrating its ability to complement experimental results. In biochar, the adsorption properties of seaweed-derived biochar,^201^ and the optimization of pollutant retention time,^202^ has been some of the studies conducted. The need for designer hydrochars with applications in energy storage, catalysis, agriculture, and oil and gas industries calls for a strong effort on the development of atomistic models and new simulation techniques for porous carbon materials.

### Fit-for-purpose biocrude oils

Biocrude is a thick bitumen-like fluid with a molecular weight profile higher than the one for bio-oils from pyrolysis due to repolymerization of light fragments during HTP. Unlike hydrochar, biocrude oils are produced in higher yields through HTL conditions. Typically, two fractions can be differentiated in biocrude oil: light oil (sometimes also named bio-oil, not to be confused with bio-oil from pyrolysis) and a heavy fraction known as heavy oil. The composition of both fractions depends on the original biomass feed, and on whether the HTL is performed in isothermal or fast conditions (Table 3). Ketones, phenols, furan, and benzene derivatives are found in both isothermal and fast HTL of polysaccharides. Indanone derivatives are present only in isothermal HTL, while acids, alcohols, esters, and hydrocarbons are present only in fast HTL.^161^ In the case of processing nitrogen-rich biomass materials like chitin, fast HTL yields pyrimidines, pyrroles, pyridinones, pyridine, amides, and triazoles, whereas isothermal HTL yields pyrrolidinediones, pyrroles, pyridines, indoles, and pyrazines.^162^

Biocrude’s composition also depends on the presence of acidic and alkaline species^203^ and extraction solvents. For instance, alkaline additives decrease the production yield,^163^ while both the elemental composition and the yield vary with the use of toluene, dichloromethane, and acetone as extraction solvents.^204^ In terms of quality, however, solvent-free HTL gives higher-quality biocrude despite the lower yield.^205^

While the production of biocrude oils and their experimental analyses are well documented, very few molecular models of biocrude oil currently exist. High molecular weights and viscosities typically exhibited by individual molecular components limit the use of gas chromatography-mass spectrometry (GC-MS) analyses, and pyrolysis-GC-MS is required. Considering these challenges in characterization of biocrude oils, there is only one molecular model reported in the literature, up to date. This is a molecular model derived from the characterization of a biocrude oil produced by HTL of microalgae.^206^ The model enabled a DFT study of the model molecules to provide information on their global and local reactivity using conceptual DFT reactivity descriptors and inferring to the tendency of biocrude to undergo oxidative aging or hydrothermal upgrading. These processes are key to design fit-for-purpose biocrude oils.^206^

Complementarily, full-atomistic MD simulations attempt to understand nanoaggregation, and colloidal properties arising from nanoscale features, i.e., heavy-oil emulsions.^207^ However, the study of bitumen-like materials requires more accelerated MD methods and new CG modeling techniques. For example, in a recent MD study of fossil asphaltenes nanoaggregation in toluene and heptane,^208^ a long simulation time of 0.5 μs still failed to reach an equilibrium state. Subsequently, the aggregation of asphaltenes was investigated using CG methods and the resultant mixture comprised nanoaggregates that were neither consistent nor reproducible in size.^209^

Finally, although liquefaction usually occurs at higher temperatures, biocrude oils are also formed during HTC.^210^ These HTC-derived biocrude oils have been characterized from fish and shrimp waste.^210^

### Biobased molecules and quantum dots

The aqueous phase contains water-soluble molecules, nutrients, and carbon nano- and micro-particles that can be extracted and isolated for further processing and utilization. It can also be valorized in agricultural applications, or within the HTP process itself.^141^

Table 4 summarizes some of the platform molecules and carbon nanoparticles identified in the aqueous phase derived from HTC. For example, HTC of model biomass components like sugars and polysaccharides at different temperatures and retention times produces furfural, hydroxymethylfurfural (HMF), acids, and aldehydes in the aqueous phase,^159^ while HTC of glucose and xylose produces 4-oxopentanoic acid (also known as levulinic acid), HMF, and formic acid.^160^ Fibers of spent sugar beets produce high-value platform molecules like glucose, fructose, sucrose, HMF, furfural, formic acid, levulinic acid, and acetic acid.^192^ In addition, functionalized carbon nanoparticles with low cytotoxicity and photoluminescent properties can also be obtained from chitosan under mild conditions and applied as a bioimaging material.^166^ Nevertheless, the yield of these carbon nanoparticles is only 7.8%.

In the case of HTL, Table 5 summarizes the platform molecules and carbon nanoparticles characterized in the aqueous phase. The composition and concentration of the molecular species characterized in HTL slightly differs from the aqueous phase in HTC. When it comes to algae, the yield of these water-soluble compounds decreased at increasing temperatures. In fact, there is a decrease in the yield of up to 10% when processing proteins at higher temperatures and above 30% when processing polysaccharides.^168^

Like in HTC, the aqueous phase from HTL also contains carbon nanoparticles and polymer nanoparticles that behave as quantum dots. In fact, HTL of grass produces water-soluble, nitrogen-doped, carbon-rich, photoluminescent polymer nanoparticles,^152^ while carbon nanoparticles were produced from a mix of glucose and glycine.^211^ Most of these nanoparticles are carbon-rich but nitrogen-doped, and they all exhibited quantum dot properties. Biobased carbon quantum dots are used for imaging of cells with low cytotoxicity^141^ and therefore have applications in biological labeling, disease diagnosis, and biosensors.^166^ In case of polymer nanoparticles, their quantum dot behavior makes them useful as fluorescent probes for Fe(III) detection in aqueous solution.^211^ Despite all these potential applications, there is still a lack of computational studies, and only very few molecular models are available. The computational studies of carbon quantum dots are mostly based on DFT and time-dependent DFT. Functionals like B3LYP, CAM-B3LYP, and wB97XD have been used to study the electronic structure of carbon nanoparticles from fruit waste,^212^ and the results are still a long way off the experimental data. New dispersion-corrected functionals are expected to produce more accurate results for biobased carbon quantum dots.^213^ Beyond DFT, there is a lack of MD simulations over 100 atoms in size, and it is still challenging to achieve high accuracy without excessive computational time.^214^ This lack of computational work clearly points out the challenges in modeling nanoparticles and the need for new ML-based approaches that provide alternatives for predictive simulations.

## The role of multiscale modeling, AI, and ML on biomass valorization

**Figure 7:**
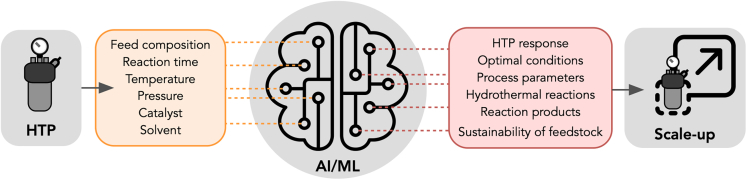
Illustration that represents the current used of AI and ML methods in HTP to support the scale-up of hydrothermal processes The flow diagram indicates the HTP data that can be used in the training of AI/ML models and the data that can be predicted to support the scale-up.

Beyond DFT and MD, the development of AI and ML methods in combination with the use of graphic processing units to accelerate high-throughput computing is changing different areas of science and technology, including molecular and materials discovery. In the case of HTP, ML algorithms are being used to predict materials properties, production yields, and reaction mechanisms, as well as to identify optimal process parameters, and suitability of feedstock to produce biocrude oils.^16^^,^^17^ The advances enabled by AI and ML are especially relevant for finding optimal processing conditions and for eventually scaled-up HTP processes (see Figure 7). However, the complex reactions that take place in HTP and the different partitioning of chemical species between solid and liquid phases during the process make mechanistic modeling extremely challenging, and challenges remain ahead.^18^

ML force field models^215^ have begun to replace *ab initio* simulations by predicting forces directly from atomic structures, and novel benchmark suites for ML-based MD simulations that implement new design evaluation metrics are being developed to increase the accuracy of these simulations.^216^ Given the exponential growth of ML models and AI techniques for molecular discovery, this is clearly just the beginning. Applying methods that have been proved successful in other areas to biobased molecules and materials is expected to revolutionize the development of nature-inspired computational materials for a circular economy. Examples are the use of active learning for free energy calculations,^217^ efficient analysis of high-throughput nanopore data,^218^ chemical dynamics simulations of interfacial systems,^219^ or physics-informed ML models.^220^

On the biomass conversion processes, only few studies have taken advantage of the capabilities of high-performance computing and computational modeling and atomistic simulations.^221^ More work has been done on the material design phase, where computational methods have played an important role in understanding the chemical and mechanical transformation of the biopolymers that constitute biomass. They have also been used in the prediction and study of physicochemical properties of both raw materials and biobased products, which is crucial for developing nature-inspired computational materials with wide ranging applications such as energy storage,^222^ agriculture,^141^ sensors,^48^ drug delivery,^47^ or tissue engineering.^223^ At the nanoscale, molecules that contain nitrogen atoms in their structure, such as proteins or some polysaccharides like chitin, are of special interest, and therefore the role of nitrogen has been the target of computational studies connected to the development of biobased materials. Nitrogen doping is used to tailor physicochemical properties of carbon functional materials, providing oxidation stability, additional thermal and electrical conductivity, and catalytic activity.^224^^,^^225^ Therefore, naturally occurring nitrogen in biomass arises as a key feature for applications, and its role needs to be better understood through fundamental research in the future.

**Figure 8:**
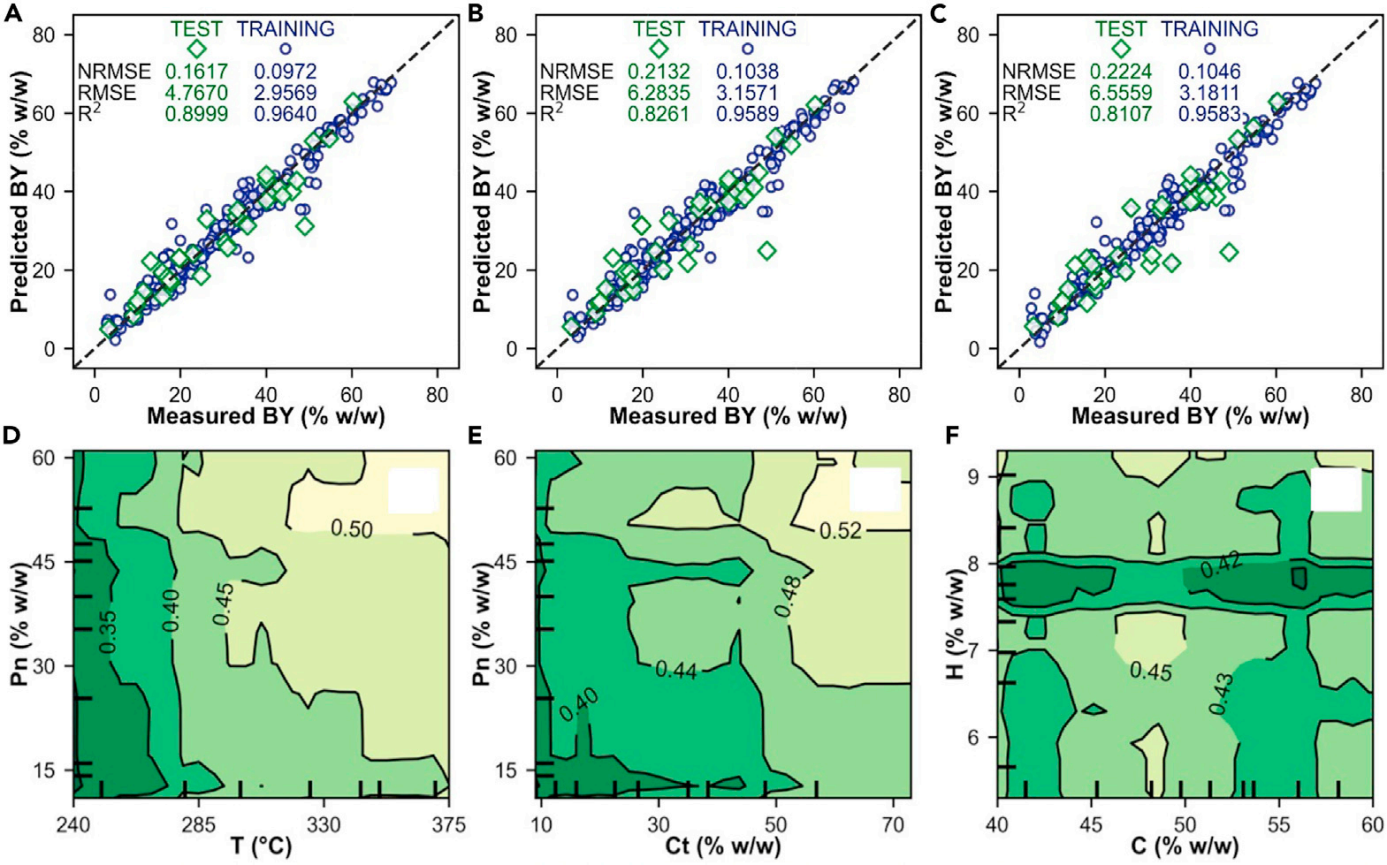
Distribution of the prediction of biocrude oil yields from HTL of wet biomass and wastes with XGBoost in Figures 8A–8C Adapted with permission from Ref.^155^ Two-dimensional predictions using partial dependence of the XGBoost predictions for biocrude yield: Figure 8D, temperature (T) versus protein content (Pn); Figure 8E, carbohydrate content (Ct) versus protein content (Pn); and Figure 8F, carbon content (C) versus hydrogen content (H). Adapted with permission from Ref .^228^

Several ML algorithms and neural networks have been applied at different stages of HTP and in the study of biomass and biobased materials. Artificial neural networks, support vector-regression, random forest algorithms, and K-nearest neighbors algorithms have been used for the prediction of HTC reaction kinetics in cellulose, poplar, and wheat straw processing.^18^ Random forest algorithms and support vector-regression have been proved successful in predicting yield, higher heating value, energy recovery efficiency, and energy densification of HTC and pyrolysis in the production of hydrochar and biochar, respectively.^226^ Multilayer perceptron artificial neural network, as well as extreme gradient boosting (XGBoost), was used in the prediction of lignocellulosic biomass conversion during HTC. These methods showed that the conversion was mostly sensitive to temperature, time, and moisture, for a range of conditions applied. According to XGBoost none of the parameters were negligible, although operating conditions were more influential, followed by lignin content.^227^ However, no experimental validation was carried out, which is a recurrent drawback for most of the ML studies available so far. XGBoost was also used in the prediction of biocrude oil yields from HTL of wet biomass and wastes,^228^ and while the accuracy of the model was overall acceptable (Figure 8), the study also lacked experimental validation. Differently, an ML study with experimental validation used gradient boosting regression, random forest algorithms, and decision regression tree algorithms to study HTL biocrude oil production.^229^ Random forest algorithms and gradient boosting regression were also used in the prediction and optimization of biocrude oils from HTL of algae.^17^ In this case, there was also experimental validation, and it was found that gradient boosting regression performed better than random forest algorithms for both single-task and multi-task prediction. Nevertheless, from the different studies available in the literature, random forest algorithms seem to be the best model for multi-task prediction when biocrude oil yield, nitrogen content, and energy recovery are the chosen variables.^17^

In the case of hydrochar, deep neural networks have been used in multi-task prediction of hydrochar properties.^230^ The results showed that both operational conditions and feedstock compositions were relevant to predict hydrochar’s properties and its carbon capture and storage ability, but without experimental validation available. In a similar way, multilayer preceptor artificial neural networks were used to predict the nitrogen content in hydrochar produced from sewage sludge.^231^ Results showed that the most relevant variables to consider were the temperature and the content of nitrogen, carbon, volatiles, and fixed carbon in the sewage sludge. In fact, the content of nitrogen in hydrochar decreases with increasing temperature and the model was validated using previous experimental reports. Given the impact that nitrogen has in the properties of biobased materials and their applications, this type of insight is especially useful. Moreover, the use of data mining and generative neural networks like those applied in zeolites could be potentially useful to design porous hydrochars in the future.^232^

The effect of catalysts and solvent on HTL was also studied both experimentally and with the use of ML models.^233^ Results revealed that the yield of biocrude oil increases with the use of alkaline catalysts rather than acid catalysts.

Carbon quantum dots have also been the object of ML studies. In fact, different ML techniques were used in one experiment to investigate the origin of the photoluminescence mechanism.^234^ Different algorithms were applied and were found to be more useful in some respects than others. While principal component analysis was used to choose the best excitation wavelength, non-negative matrix factorization (NMF-ARD-SO) was advantageous in the study of the photoluminescence mechanism.

## Conclusions and outlook

**Figure 9:**
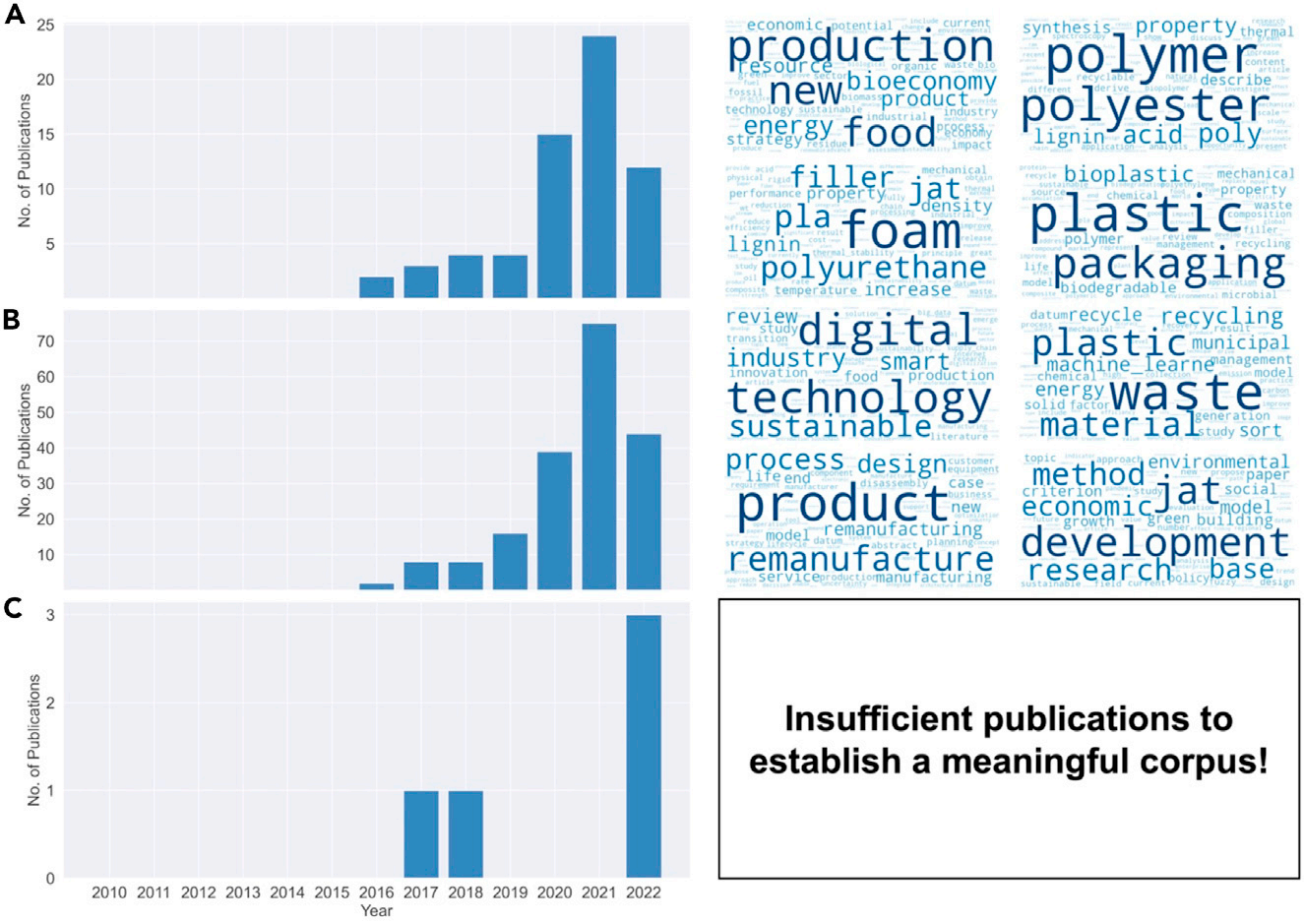
Mining journal publication titles and abstracts from the scholarly database, Lens.org, using search terms of Figure 9A (circular economy) AND (biobased materials), Figure 9B (circular economy) AND ((machine learning) OR (artificial intelligence)), Figure 9C ((machine learning) OR (artificial intelligence)) AND (biobased materials). Four topic models per search and their corresponding wordclouds were then generated using term-frequency - inverse document-frequency (tf-idf) with non-negative matrix factorization (NMF).

Available biomass waste offers an opportunity for producing nature-inspired biobased materials that provide similar or better performance to petroleum-derived ones. Carbon mining in those wastes will reduce the current pressures on natural resources, while contributing to a circular economy. Advances in materials processing, manufacturing, and characterization, as well as the use of cloud supercomputing, high-throughput computational modeling, AI, and ML, are supporting this new mining of materials and waste valorization. The rising importance of such integrated research is playing out in the scientific realm, and there is a clearly increasing trend in related scientific publications, yet with noticeable potential gaps in applying AI and ML in combination with biobased materials (see Figure 9).

While there is no discordance that billions of tons of biomass waste are generated every year, the exact amount is not clear. Standardized evaluations of biomass resources available are needed to make informed decisions. Furthermore, the lack of a well-defined criterion for classifying biomass types makes the comparison of available data almost impossible for now, hampering data-driven conclusions. Also in this case, standards are needed to better facilitate an analysis of the current landscape and resources availability and composition.

Some technical bottlenecks still hamper an industrial transition to biobased materials, including logistics, handling and storage costs, physical and chemical processes efficiency and optimization, materials extraction, catalysis, and depolymerization. They need to be addressed to guarantee sustainable and cost-effective technologies. The same applies to materials characterization. Although there are countless techniques available in materials science and engineering, adapting them to characterize biomass waste and biobased materials requires additional developments.

A better understanding, at a molecular level, of the physicochemical properties of both raw materials and biobased products, as well as of transformation mechanisms and processes, will lead to improvements in the methods and in the control of the products that are obtained. Computational tools can assist, being more efficient and less costly than experiments. However, more complete models of biomass and biobased materials are needed to enable predictive computational simulations. For example, the development of better models for hydrochar and biocrude oils will provide further insights on composition-process-structure-property relationships. Further research with AI and ML is required for the prediction and optimization of HTP to help its scaling-up and commercialization. Up until now, most studies use single-target prediction or small datasets or are time-consuming with a high computational cost. The availabilities of limited datasets and the lack of experimental validation are very common challenges in the use of ML. Furthermore, as stated by Peng et al.,^235^ breakthroughs in molecular and materials discovery require meaningful outliers to be identified in existing trends. Thus, data-driven approaches that reduce cognitive overload and biases, while establishing atomistic understanding that is transferable across the chemical space, are needed.

If we are meant to rethink our material sources and products design, from systems level to the nanoscale, we must valorize the extensively available biomass wastes, as new carbon mines, and we must mimic nature’s efficiency and ability to design out waste. This is already changing the way we think about materials flows, and it is initiating a shift from the traditional linear economy of materials utilization to a new biobased economy, which is an enabler of a wider circular one. However, more research, policies, and investment are still needed, and the coming years are crucial to shape a sustainable development for the generations to come.
